# The Soybean Peptide Vglycin Preserves the Diabetic β-cells through Improvement of Proliferation and Inhibition of Apoptosis

**DOI:** 10.1038/srep15599

**Published:** 2015-10-29

**Authors:** Hua Jiang, Yuxing Tong, Dongjing Yan, Shaohui Jia, Claes-Goran Ostenson, Zhengwang Chen

**Affiliations:** 1 Key Laboratory of Molecular Biophysics of Ministry of Education, School of Life Science and Technology, Huazhong University of Science and Technology, Wuhan 430074, P.R. China; 2 Institute of Immunology and the CAS Key Laboratory of Innate Immunity and Chronic Disease, School of Life Sciences and Medical Center, University of Science and Technology of China, Hefei 230027, China; 3 Department of Biochemistry and Molecular Biology, Hainan Medical College, Haikou 571199, China; 4 College of Health Science, Wuhan Sports University, Wuhan 430079, P.R. China; 5 Department of Molecular Medicine, Karolinska Hospital, Stockholm, Sweden

## Abstract

Replenishment of insulin-producing pancreatic β-cells would be beneficial in diabetes. The number of β-cells is maintained primarily by self-neogenesis to compensate for β-cell failure, loss or dedifferentiation. We present here a polypeptide vglycin, which was isolated and purified from germinating pea seeds. Vglycin exhibited positive effects in our diabetic models by promoting the proliferation and suppressing the apoptosis and dedifferentiation of β-cells. Vglycin promoted the restoration of β-cells in both young streptozotocin (STZ)-induced type 1 diabetic SD rats and in aged high-fat diet with (or without) STZ-induced type 2 diabetic C57BL/6 mice. We demonstrated that vglycin triggers this positive signaling by activating the insulin receptor and corresponding transcription factors. Impaired insulin sensitivity and glucose tolerance in aged T2DM mice were dramatically improved after long-term vglycin treatment, consistent with the altered level of inflammatory factor IL-1β/6. In addition, energy expenditure and body weights were significantly decreased in the mouse models after vglycin therapy. These results provide insight into the protective effects of vglycin on ameliorating β-cell function in standing glucolipotoxicity. Thus, vglycin may represent a new therapeutic agent for preventing and treating diabetes by replenishing endogenous insulin-positive cells.

Diabetes, a heterogeneous disorder with complex etiologies, is characterized by abnormal carbohydrate metabolism caused by insufficient insulin release^1^. Diabetes has become one of the most serious threats to human health. More than 380 million people worldwide live with diabetes, and the number is predicted to reach 471 million by 2035^1^,^2^,^3^. Life-long injection with exogenous insulin is required in type 1 diabetes, which is primarily caused by autoimmune β-cell destruction and consequent deficiency^4^. T2DM, the predominant type of diabetes, is characterized by impaired peripheral insulin sensitivity and glucose tolerance, ultimately leading to β-cell failure and diminution or dedifferentiation. These β-cells subsequently fail to secrete sufficient insulin to maintain normoglycemia. β-cells enhance insulin secretion to compensate and expand when persistently exposed to a hyperglycemic circumstance, which ultimately leads to β-cell exhaustion^5^,^6^. Insulin injection or administration of other antidiabetic drugs can alleviate the disease to some extent. However, therapies that contribute to β-cell replenishment by reducing β-cell death and increasing functional β-cell mass in diabetic patients would be the best way to control hyperglycemia^7^. Although the primary causal factors differ in T1DM and T2DM, patients with either type would benefit from therapies that improve β-cell mass and function. Numerous studies have indicated that the majority of neogenesis in β-cells is derived from self-duplication and redifferentiation from dedifferentiated β-cells^8^,^9^,^10^. Therefore, the regeneration of β-cells occurs via at least two pathways: self-replication and conversion from other cell types. The replication rate of β-cells is extremely low in both adult rodents and humans but is elevated in response to challenges such as hyperglycemia, pancreatic injury, insulin resistance and other extreme stress challenges. “Proliferation” can also occur by lowering the rate of β-cell apoptosis or death^11^.

As a mitogen of β-cells, glucose enhances β-cell replication in the presence of glucokinase^12^,^13^. In addition to glucose, hormones such as insulin, prolactin, and the incretin family of polypeptides have also been demonstrated to promote β-cell regeneration and function^11^. Conversely, chronic metabolic stresses such as aging, obesity and overnutrition can result in the failure of β-cell function and mass^14^. Many studies have examined the roles of transcription factors such as Pdx1, MafA, Nkx6.1, FoxO-1 and Neurogenin3 during the progression of metabolic challenge^5^,^15^,^16^. Under the stresses described above, signals triggered by extracellular agents contribute to the survival and growth of β-cells at least in part by activating the insulin receptor (IR)/Akt signaling pathway. Insulin or IGF-I signaling is necessary for the correct functioning and maintenance of β-cell mass^17^,^18^,^19^,^20^. Erk, a critical downstream kinase, plays a key role in regulating cell proliferation.

Previously, we reported that vglycin normalizes fasting plasma glucose (FPG) levels in young type 2 diabetic Wistar rats by improving insulin sensitivity, glucose tolerance and islet restoration, while vglycin did not have toxic effects on organ functions of normal BALB/c mice^21^. Here, we demonstrate that vglycin preserves β-cells in both T1DM SD rats and aged T2DM C57BL/6 mice by promoting their proliferation and suppressing their apoptosis and dedifferentiation. Immunoblotting assays revealed the molecular mechanisms of vglycin in these processes. Overall, our results provide direct evidence for vglycin as a potential antidiabetic agent, although the precise mechanisms remain to be elucidated.

## Results

### Vglycin normalizes plasma glucose levels and preserves islets and β-cells in juvenile T1DM SD rats

**Figure 1:**
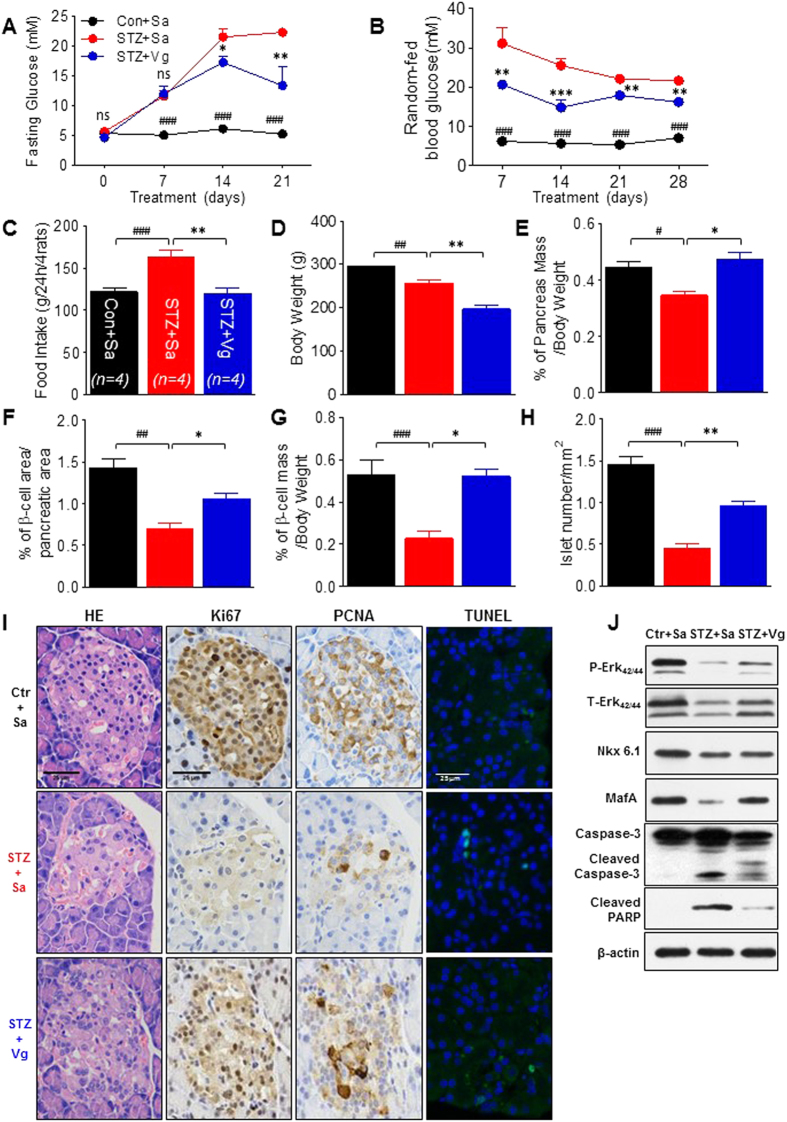
The soybean peptide vglycin preserves pancreatic islets in T1DM SD rats. Effects of vglycin on fasting plasma glucose (FPG) (**A**), random-fed plasma glucose (**B**), food intake (**C**) and body weight (BW) (**D**) in T1DM SD rats. Quantification of the proportional pancreas mass (**E**), β-cell area (**F**) and β-cell mass (**G**) as well as the number of islets (**H**). Representative images of whole sections stained with hematoxylin and eosin (**H,E**) and immunostained with anti-Ki67/PCNA antibodies (brown staining) are shown. Islet cell apoptosis measured by immunofluorescence TUNEL staining. Scale bar: 25μm (**I**). Pancreas extracts were immunoblotted (IB) with anti-P/T-Erk_42/44_, NKX6.1/MafA, and caspase-3/PARP antibodies as well as anti-β-actin antibody; immunoblotting was performed at least in duplicate. Representative bands are shown (**J**). Data are presented as mean ± SEM values, n = 4 rats per group, three pancreatic sections per rat. *the STZ + Sa group *vs.* the STZ + Vg group. ^#^the Con + Sa group *vs.* the STZ + Sa group. *,**p < 0.05, 0.01. ^#,##,###^p < 0.05, 0.01, 0.001. ns, no significance.

We previously demonstrated that vglycin has beneficial effects in young T2DM Wistar rats^21^. To examine the protective effects of vglycin in the diabetic pancreas, we first investigated the effects of vglycin on plasma glucose control in a T1DM animal model. Administration of vglycin at a dose of 80 mg/kg/day to T1DM SD rats caused a significant decrease in both FPG and random-fed blood glucose (Fig. 1A,B) during the four-week vglycin treatment period. Both food intake during treatment and body weight after treatment were markedly reduced in treated rats compared with untreated matched controls (Fig. 1C,D). We next examined whether vglycin affects the pancreatic islets of young T1DM SD rats. We first compared the pancreas mass in terms of unit body weight. As shown in Fig. 1E, the pancreas masses of the vglycin-treated group was significantly increased (p < 0.05). Pancreas sections were analyzed by histomorphometry. Insulin immunolabeling indicated that both β-cell area and β-cell mass were increased in the vglycin-treated group compared to the controls (p < 0.05) (Fig. 1F,G). Furthermore, the number of islets was dramatically increased in the vglycin-treated group (p < 0.01) (Fig. 1H). We then investigated the proliferation of total ß- cells following immunostaining of pancreas sections with anti-Ki67/PCNA antibodies. Immunofluorescence TUNEL staining was used to label apoptotic cells in the islets. As shown in Fig. 1I, the density of the positive Ki67/PCNA immunostaining area was greater in the vglycin-treated group than the control group. There were much fewer TUNEL-positive/ total ß-cells in the vglycin-treated group than the control group (Supplementary Fig. 3A). Consistent with the above observations, immunoblot analysis of pancreas extracts revealed that the levels of Erk and the β-cell markers Nkx6.1 and MafA were slightly upregulated in vglycin-treated rats (Fig. 1J). Moreover, as indicated in Fig. 1J, the levels of cleaved caspase-3 and cleaved PARP were reduced in the vglycin-treated group.

**Figure 2:**
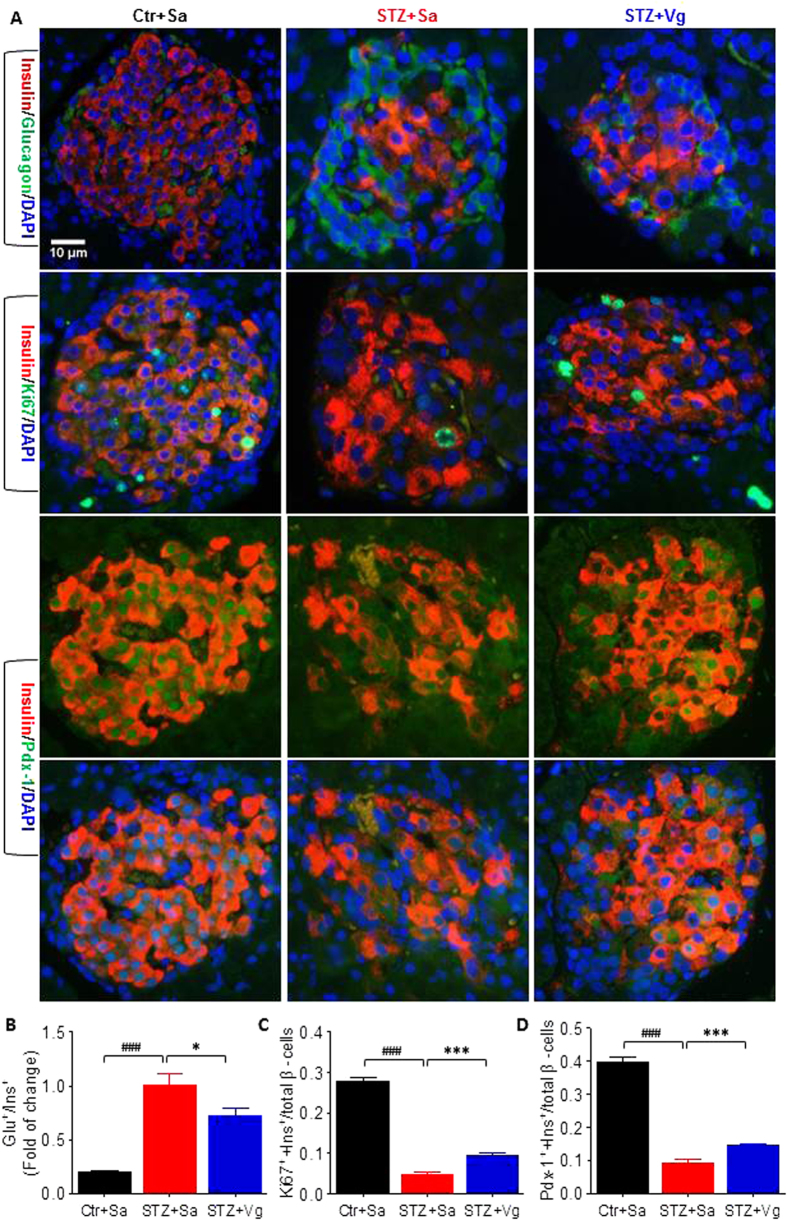
The soybean peptide Vglycin preserves pancreatic β-cells in T1DM SD rats. Representative images of double immunostaining for insulin (red)/glucagon (green), insulin (red)/Ki67 (green) and insulin (red)/Pdx-1 (green). Nuclei were stained with DAPI (blue). Scale bar: 10μm (**A**). Quantification of the ratios of glucagon-positive cells to insulin-positive cells (**B**), insulin and Ki67 double-positive cells to total ß-cells (**C**) and insulin and Pdx-1 double-positive cells to total ß-cells (**D**). Data are presented as mean ± SEM values. n = 4 rats per group, three pancreatic sections per rat. At least 20 islets containing approximately 2000 cells from each pancreatic section were analyzed. *,***p < 0.05, 0.001. ^###^p < 0.001.

We next directly determined if islet β-cells benefited from vglycin treatment by double immunofluorescence staining of pancreas sections. Double immunostaining for insulin and glucagon revealed that the ratio of glucagon-positive cells to insulin-positive cells was significantly lower in in the vglycin-treated group (~0.726) than the control group (~1.01) (Fig. 2A,B). Double immunostaining for insulin and Ki67 revealed that the ratio of cells positive for both insulin and Ki67 to total ß-cells was significantly higher in the vglycin-treated group (~0.0959) than the control group (~0.0485) (Fig. 2A,C). Double immunostaining for insulin and Pdx-1 revealed that the ratio of cells positive for both insulin and Pdx-1 to total ß- cells was significantly higher in the vglycin-treated group (~0.145) than the control group (~0.0923) (Fig. 2A,D).

### Chronic vglycin treatment ameliorates metabolic stress in middle-aged T2DM mice

**Figure 3:**
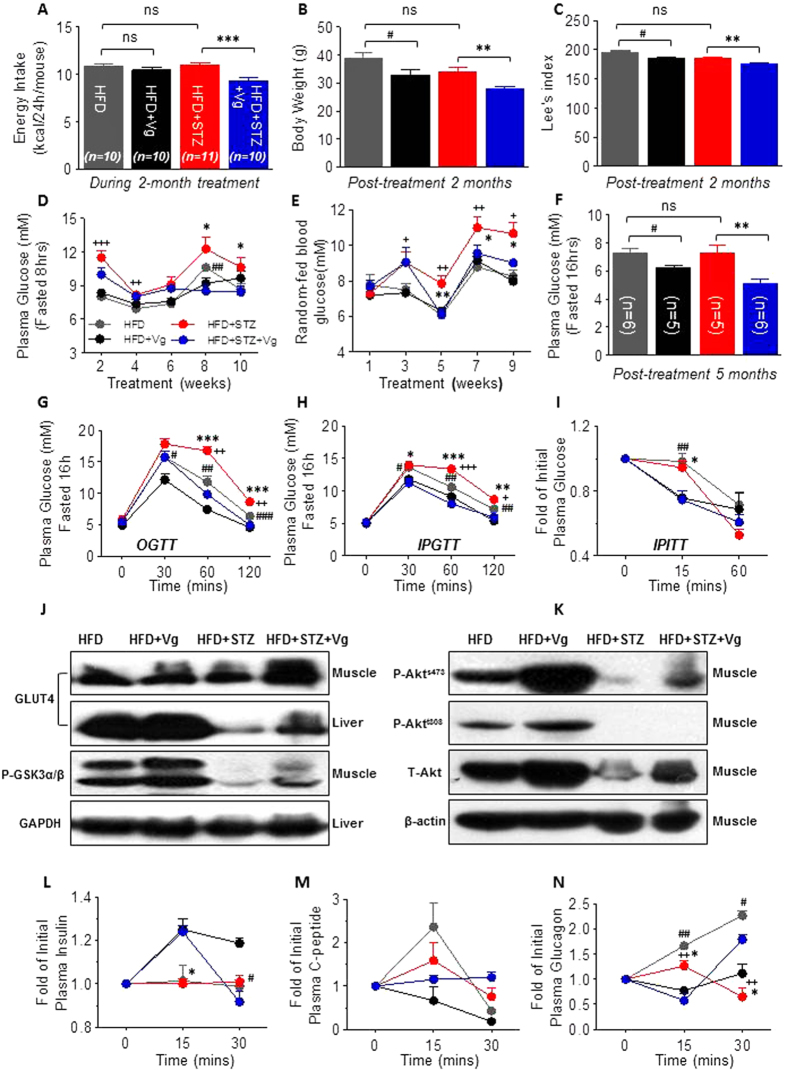
Chronic vglycin treatment benefits middle-aged T2DM mice. Effects of chronic vglycin treatment on average energy intake (**A**), body weight (BW) (**B**) and Lee’s index (**C**) during or after 2 months of vglycin treatment. Fasting plasma glucose (FPG) and random-fed plasma glucose were measured every other week from the beginning of vglycin treatment (**D**,**E**), n = 10–11 for each group (**A–E**). After a 5-month treatment, a final fasting plasma glucose measurement was taken (**F**). OGTT, IPGTT and IPITT were conducted at the indicated times (**G–I**), n = 5–6 for each group (**F–I**). Equal amounts of protein extracted from isolated liver or muscle tissue were subjected to immunoblotting analysis. Representative immunoblots for GLUT4, p-GSK3α/β and GAPDH (**J**) as well as p-Akts473, p-Aktt308, total Akt and β-actin (**K**) are shown. Each immunoblot was performed in triplicate. Blood samples were collected from the tail veins of mice at the indicated time points during the glucose challenge and subjected to ELISA. The levels of initial plasma insulin, plasma C-peptide and plasma glucagon in each group were calculated and expressed. n = 3–4 for each group (**L–N**). Data are presented as mean ± SEM values. *the HFD + STZ group vs. the HFD + STZ + Vg group. ^#^the HFD group vs. the HFD + Vg group. ^+^the HFD + STZ group vs. the HFD group. *,**,***p < 0.05, 0.01, 0.001. ^#,##,###^p < 0.05, 0.01, 0.001. ^+,++,+++^p < 0.05, 0.01, 0.001. ns, no significance.

Obesity, aging and T2DM negatively affect the survival of β-cells^11^,^22^. Having demonstrated protective effects of vglycin in juvenile T1DM SD rats, we examined the ability of vglycin to exert similar effects on aged T2DM models established by maintaining mice for 12 weeks on a standard high-fat diet (HFD) alone and/or following multiple low doses of STZ injections. When the mice were 28 weeks old, we analyzed the average energy expenditure per 24 hr of each mouse during the previous 8-week vglycin-treated period, the real-time body weight (BW), and the Lee’s index. Caloric consumption was dramatically restricted in the HFD + STZ + Vg group compared to the HFD + STZ group (p < 0.001), but we did not observe a significant decline in the HFD + Vg group (p > 0.05) (Fig. 3A). Compared to their respective controls, BW and the Lee’s index were significantly reduced in both vglycin-treated groups (p < 0.05, 0.01) (Fig. 3B,C). These results suggest that vglycin may potentiate anti-obesity effects. FPG (8-hr fasting) or random-fed plasma glucose was detected once every other week during the first ten-week vglycin treatment period. As shown in Fig. 3D,E, after a 5- to 6-week vglycin treatment period, random-fed plasma glucose and FPG levels in the HFD + STZ + Vg group both tended to be normal following a significant drop compared to the HFD + STZ group (p < 0.05, 0.01). However, we did not observe any obvious changes in plasma glucose levels in the HFD + Vg group, except for an occasional decrease in FPG (p > 0.05, < 0.01) in the 8^th^ week of vglycin treatment. The last FPG test was performed after a 16-hour fasting period after 40 weeks of vglycin treatment, which revealed that FPG was significantly lower in both vglycin-treated groups (p < 0.05, 0.01) (Fig. 3F). We stopped vglycin therapy and performed glucose and insulin tolerance tests after 40 weeks of treatment. Compared to their matched controls with an abnormal glucose profile, both vglycin-treated groups were glucose tolerant (p < 0.05, 0.01, 0.001) (Fig. 3G,H, supplementary Fig. 4), and FPG tended to be lower in the treated mice (Fig. 3F). Consistent with this result, liver and muscle GLUT4 content and muscle phosphorylated GSK3α/β levels were significantly upregulated in both vglycin-treated groups (Fig. 3J). We thus determined if the improved glucose tolerance in the vglycin-treated groups was a result of improved insulin sensitivity. An insulin tolerance test (ITT) revealed that the ability of insulin to eliminate glucose was improved in vglycin-treated mice (Fig. 3I). Accordingly, muscle phospho-Akt (Ser473/Thr308) levels in the HFD + Vg group and phospho-Akt (Ser473) levels in the HFD + STZ + Vg group were significantly upregulated compared to their controls (Fig. 3K). Taken together, these results suggest that chronic vglycin treatment ameliorates metabolic stress in middle-aged T2DM mice by improving glucose and insulin tolerance to control obesity and normalize plasma glucose, consistent with our previous findings in young T2DM Wistar rats.

On the basis of our metabolic studies, we determined if vglycin directly improves diabetic islets in these models. Hormones such as insulin, C-peptide and glucagon are secreted by islet β-cells and α-cells after glucose loading *in vivo*. Plasma insulin levels are governed by a combination of the insulin secretory capacity of individual β-cells and the number of β-cells. In this study, we performed an analysis of glucose-stimulated insulin, C-peptide and glucagon secretion by ELISA. As shown in Fig. 3L, after glucose injection, plasma insulin levels were enhanced at 15 min and 30 min in both vglycin-treated groups (p < 0.05), whereas no significant changes in C-peptide levels were observed in either of the vglycin-treated groups (p > 0.05) (Fig. 3M). Furthermore, glucose-stimulated glucagon secretion was significantly suppressed at 15 min and 30 min in the HFD + Vg group (p < 0.05, 0.01). Glucagon levels in the HFD + STZ + Vg group during glucose challenge were reduced at 15 min (p < 0.05) but enhanced at 30 min (p < 0.05), potentially indicating improved balance of plasma glucose (Fig. 3N). In summary, we determined that chronic vglycin treatment indirectly and directly benefits islets.

### Chronic vglycin treatment improves the inflammatory milieu and islets in aged T2DM mice

**Figure 4:**
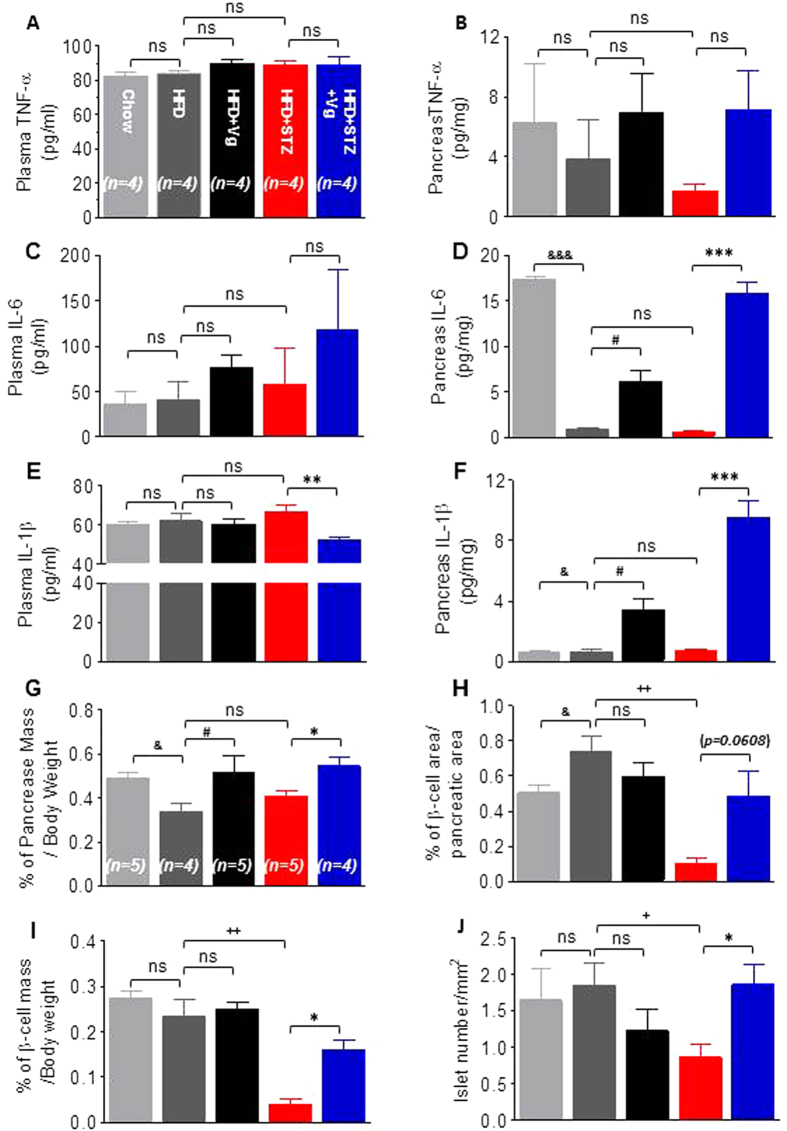
Chronic vglycin treatment ameliorates the inflammatory milieu and benefits islets in middle-aged T2DM mice. Plasma levels and pancreatic content of TNF-α, IL-6 and IL-1β in each group were detected by ELISA. n = 3–4 for each group (**A–F**). Quantification of the proportional pancreas mass (**G**), β-cell area (**H**) and β-cell mass (**I**) as well as the number of islets (**J**). n = 5–7 for each group (**G–J**). Data are presented as mean ± SEM values. ^&^the HFD group *vs.* the Chow group. ^#^the HFD group *vs.* the HFD + Vg group. ^+^the HFD + STZ group *vs.* the HFD group. *the HFD + STZ group *vs.* the HFD + STZ^+^ Vg group. *,***p < 0.05, 0.001. ^#^p < 0.05. ^+,++^p < 0.05, 0.01. ^&,&&&^p < 0.05, 0.001. ns, no significance.

Local inflammation in insulin-producing pancreatic islets resulting from immune cell infiltration and local cytokine production is closely related to the failure to maintain normal blood glucose levels^23^,^24^. These local inflammatory processes, coupled with glucolipotoxicity, result in the accelerated loss of β-cell mass via cell apoptosis or death and severely impair the insulin-producing capabilities of the remaining β-cells in T2DM patients as well as in rodent models. As previously reported in the literature, during the progression of obesity, T2DM and aging, proinflammatory cytokines such as TNF-α, IL-6 and IL-1β are elevated in both the circulation and pancreatic islets^25^. Of these cytokines, IL-1β and IL-6 are two critical regulators of islet dysfunction and destruction^26^,^27^. By contrast, emerging research also suggests that the survival of β-cells is enhanced when they are exposed to appropriate doses of IL-1β and IL-6 for a limited period^28^,^29^,^30^. In the present study, we detected levels of both of these cytokines in sera and pancreatic tissues by ELISA. We observed no significant changes in either serum or pancreatic TNF-α levels in the vglycin-treated groups compared with the corresponding controls (p > 0.05) (Fig. 4A,B). When we detected IL-6, at a low-level (~0–20 pg/mg) of IL-6, both vglycin-treated groups exhibited a significant enhancement expression of IL-6 in pancreatic tissues (p < 0.05, 0.001) (Fig. 4D), but no striking changes were detected in sera from the vglycin-treated groups (p > 0.05) (Fig. 4C). After chronic vglycin treatment, serum IL-1β levels were significantly lower in the HFD + STZ + Vg group than the HFD + STZ group (p < 0.01) (Fig. 4E). By contrast, the pancreatic IL-1β content was higher in both vglycin-treated groups than the matched controls (p < 0.05, 0.001) (Fig. 4F). Collectively, our data suggest that chronic vglycin treatment improves both circulating and local inflammatory factors under diabetic stress.

To further examine whether the survival of islets and β-cells is improved after chronic vglycin treatment in these models, we first analyzed the relative pancreas mass, β-cell area, β-cell mass and the number of islets per unit of pancreatic area. As indicated in Fig. 4G–J, compared to the HFD + STZ group, the studied parameters were all slightly or significantly increased in the HFD + STZ + Vg group (p = 0.0608; p < 0.05; 0.05; 0.05). However, compared to the HFD group, no obvious changes were observed in the HFD + Vg group (p > 0.05), except for a slight increase in the relative pancreas mass (p < 0.05). Consistent with previous results, the β-cell area was expanded in mice under the stress of the HFD diet compared to those fed normal chow (hereafter the “Chow group’) (p < 0.05) (Fig. 4H). In the present study, we observed that the relative pancreas mass was higher in the HFD group than in the Chow group (p < 0.05) (Fig. 4G). Taken together, these results suggest that chronic vglycin treatment attenuates the levels of both systemic and local inflammation and thus may directly improve the survival of islets and β-cells.

### β-cells in aged T2DM mice directly benefit from chronic vglycin treatment

**Figure 5:**
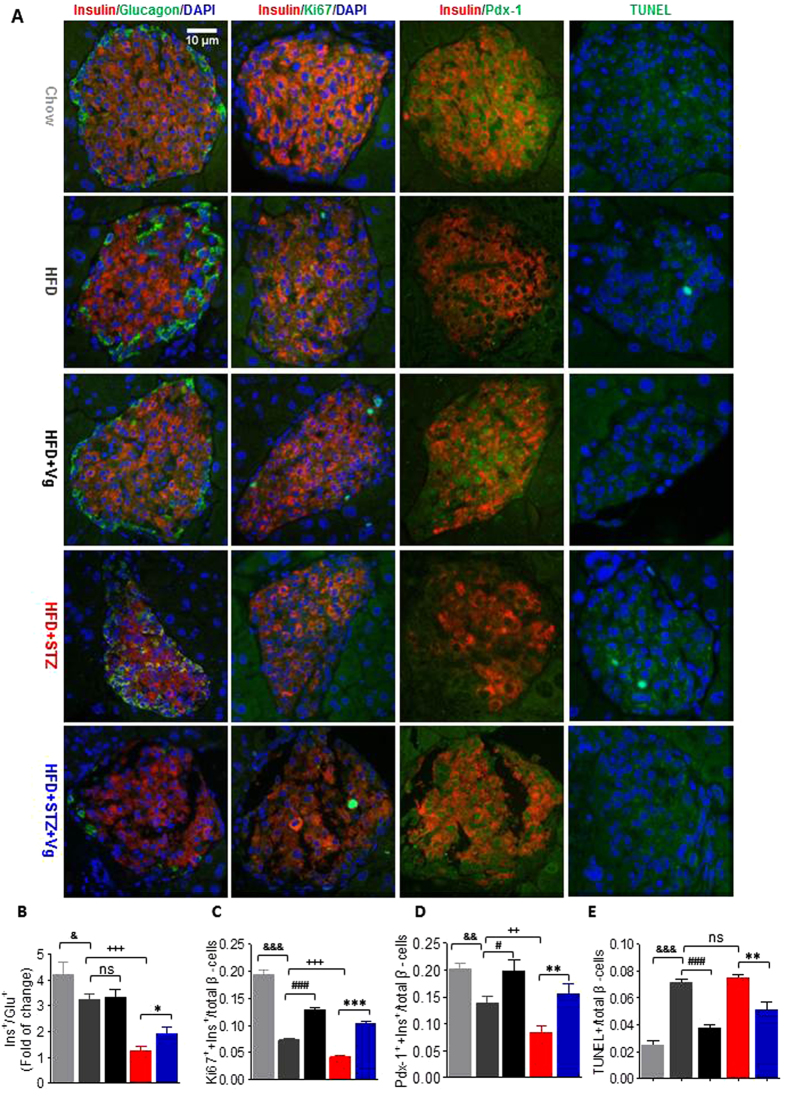
Chronic vglycin treatment directly benefits pancreatic β-cells in middle-aged T2DM mice. Representative images of double immunostaining for insulin (red)/glucagon (green), insulin (red)/Ki67 (green), insulin (red)/Pdx-1 (green) and insulin (red)/TUNEL (green). The nuclei were stained with DAPI in blue. Scale bar: 10μm (**A**). Quantification of the ratios of glucagon-positive cells to insulin-positive cells (**B**), insulin and Ki67 double-positive cells to total ß-cells (**C**), insulin and Pdx-1 double-positive cells to total ß-cells (**D**) and TUNEL positive cells to total ß-cells (**E**). Data are presented as mean ± SEM values. n = 5–6 mice per group, three pancreatic sections per mouse. At least 30 islets containing approximately 2000 cells from each group were analyzed. *,***p < 0.05, 0.001. ^#,###^p < 0.05. ^++,+++^p < 0.01, 0.001. ^&,&&,&&&^p < 0.05, 0.01, 0.001. ns, no significance.

Prompted by the above observations, we performed double immunofluorescence staining of pancreas sections to directly examine the possibility that chronic vglycin treatment improves β-cell and islet survival. As shown in Fig. 5A,B, the ratio of insulin-positive cells to glucagon-positive cells was significantly higher in the HFD + STZ + Vg group than the control group (HFD + STZ versus HFD + STZ + Vg, 1.265 versus 1.909, p < 0.05). However, compared to the HFD group, there was no significant difference in the ratio in the HFD + Vg group (HFD versus HFD + Vg, 3.248 versus 3.331, p > 0.05); instead, we observed a slight improvement of this ratio in the Chow group (HFD versus Chow, 3.248 versus 4.220, p < 0.05) and a distinct decrease in this ratio in the HFD + STZ group as expected (HFD versus HFD + STZ, 3.248 versus 1.265, p < 0.001). Double immunostaining for insulin and the cell proliferation marker Ki67 revealed that the ratio of cells positive for both insulin and Ki67 to total ß-cells was distinctly higher in both vglycin-treated groups compared to the control groups (HFD versus HFD + Vg, 0.0729 versus 0.1298, p < 0.001; HFD + STZ versus HFD + STZ + Vg, 0.0411 versus 0.103, p < 0.001). Compared to the HFD group, the ratio of cells positive for both insulin and Ki67 to total ß-cells was strikingly higher in the Chow group (HFD versus Chow, 0.0729 versus 0.195, p < 0.001) but distinctly reduced in the HFD + STZ group (HFD versus HFD + STZ, 0.0729 versus 0.0411, p < 0.001), (Fig. 5A,C). Pdx-1, the master regulator of β-cell fate, plays a critical role in pancreatic development and mature β-cell function. In the current study, we used Pdx-1 as a definitive marker of β-cells and measured the percentage of insulin and Pdx-1 double-positive cells and the number of total β-cells. Approximately 15.3% of the islet cells were double positive for Pdx-1 and insulin in the HFD + STZ + Vg group, compared to only 8.2% in the HFD + STZ group (p < 0.01). The percentages were 20.2%, 14.3% and 20.7% in the Chow, HFD and HFD + Vg groups, respectively. Compared to the HFD group, the percentages of double-positive cells were significantly higher in both the Chow and HFD + Vg groups (p < 0.01) (Fig. 5A,D). Finally, we detected the apoptotic status of ß-cells under the stresses of aging, HFD and HFD + STZ by immunofluorescence TUNEL staining. The immunofluorescence staining clearly revealed that the ratio of TUNEL positive cells to total ß-cells was significantly higher in the HFD group than in either the Chow or HFD + Vg group (HFD versus Chow, 0.072 versus 0.025, p < 0.001; HFD versus HFD + Vg, 0.072 versus 0.038, p < 0.001). By contrast, no significant increase in the ratio was observed in the HFD + STZ group (p > 0.05). Compared to the HFD + STZ group (7.5%), the percentage of apoptotic cells in the HFD + STZ + Vg group was significantly reduced (5.2%; p < 0.05) (Fig. 5A,5E). Taken together, these results indicate that under diabetic stress, chronic vglycin treatment can directly benefit β-cells, possibly by promoting their proliferation, inhibiting their conversion to other cell types and reducing their rate of apoptosis.

### Chronic vglycin treatment dramatically alters the expression of critical factors that are closely implicated in the proliferation and preservation of β-cells

To extend our findings, we explored the molecules involved in the response to chronic vglycin treatment. Vglycin shares high structural homology with PA1b and leginsulin^31^,^32^. Both PA1b and leginsulin compete with insulin or IGF for binding to a 43-kDa plant basic 7 S global protein with some structural homology to the mammalian insulin receptor^33^,^34^,^35^. In addition, emerging evidence indicates that mediators and effectors of insulin and IGF-1 signaling, including IR, FoxO1 and Akt kinases, play critical roles in β-cell growth and function^36^,^37^. Here, we determined if vglycin can function like insulin or IGF to activate the mammalian IR.

**Figure 6:**
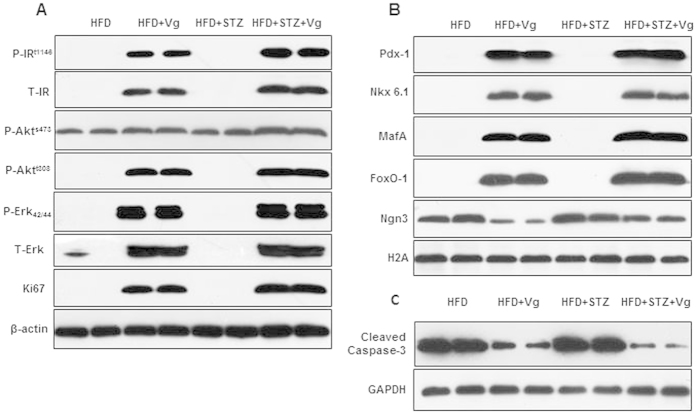
Chronic vglycin treatment dramatically alters the expression of critical factors implicated in β-cell proliferation and preservation. Pancreatic tissues were isolated from two randomly selected mice from each group. Western blot assays of the pancreatic tissues were performed using the indicated antibodies. The western blot data represent 5–6 mice per group. Critical factors that are closely related to the progression of proliferation of β-cells (**A**). Key factors that are closely associated with the characterization and maturation of β-cells (**B**). Western blots of cleaved caspase-3 and GAPDH in the pancreatic tissues (**C**). The data are representative of three independent experiments.

Strikingly, immunoblotting analysis indicated that chronic vglycin treatment induced strong expression of IR, Akt, Erk and the proliferation marker Ki67 in the pancreatic tissues of both vglycin-treated groups; however, there was nearly no expression of these proteins in the HFD and HFD + STZ groups except for Akt (Fig. 6A). To determine if β-cells were directly affected by chronic vglycin treatment, we detected markers of β-cells, such as Pdx-1, MafA and Nkx6.1, by immunoblot, which revealed that the proteins of both vglycin-treated groups were strikingly increased (Fig. 6B). FoxO1, a multifunctional protein, is necessary for maintaining β-cell function and identity during increased metabolic stress, such as aging and nutrient oversupply^38^. Neurogenin-3 (NGN-3), an endocrine progenitor marker, is highly expressed in dedifferentiated β-cells^39^,^40^. In the current study, equal amounts of pancreatic tissue were sampled, separated and analyzed by immunoblotting with primary antibodies for FoxO1 and NGN-3. As shown in Fig. 6B, we observed strong activation of FoxO1 in both vglycin-treated groups. Conversely, NGN-3 expression was significantly lower in both vglycin-treated groups compared to the controls (Fig. 6B). Taken together with Fig. 5A–D, these results indicate that chronic vglycin treatment contributes to the proliferation of β-cells by increasing replication and sustainability.

Enhanced apoptosis may contribute to the decrease in β-cells in T2DM. Apoptosis was therefore examined in pancreatic sections and tissues by immunostaining (Fig. 5A,E) and immunoblotting assays with an antibody to cleaved caspase-3. Consistent with the data in Fig. 5A,E, pancreas levels of cleaved caspase-3 were lower in both vglycin-treated groups compared to the controls (Fig. 6C).

### Vglycin contributes to proliferation and anti-apoptosis in INS-1 832/13 cells

**Figure 7:**
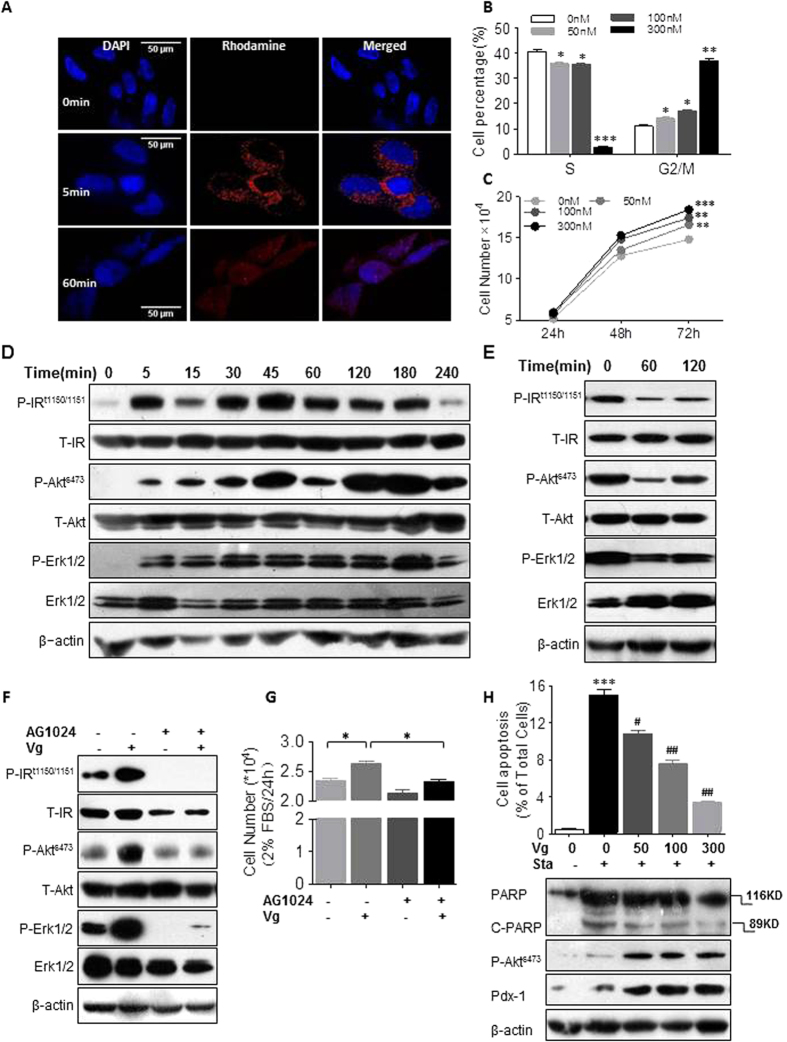
The IR/Akt/Erk pathway is involved in the proliferation of INS-1 832/13 cells in response to vglycin. Confocal microscopy of INS-1 832/13 cells pretreated with 300 nM rhodamine-labeled vglycin (red) and with DAPI (blue) at the indicated time points. Scale bar: 50 μm (**A**). INS-1 cells were serum starved for 15 hr in RPMI 1640 medium containing 2% FBS and then incubated with the indicated amounts of vglycin or without vglycin as a basal control for 24 hr. The cells were collected and subjected to flow cytometry analysis to determine the distribution of the cell cycle stages (**B**). INS-1 cells were serum starved as above and then co-cultured with the indicated amounts of vglycin or without vglycin for 24 h, 48 h and 72 h. Cell proliferation was measured by cell counting kit 8 (CCK-8). n = 9 for each group per independent experiment (**C**). INS-1 cells were serum starved and then co-cultured with 300 nM vglycin for the indicated times or without vglycin. Cell extracts were immunoblotted with the indicated primary antibodies (**D**). INS-1 cells were pretreated with 300 nM vglycin for 5 min, then removed the medium. The cells were continuously co-cultured in normal medium for the indicated times or analyzed at 0 min. Cell extracts were immunoblotted as described above (**E**). After serum starvation for 15 hr, INS-1 cells were pretreated with 0.2 μM AG1024 for 4 hr and then exposed to 300 nM vglycin in starvation medium for 24 hr. Western blots of the cell extracts were performed (**F**), and proliferation was measured by CCK-8 (**G**). Cells were starved overnight and pretreated with 400 nM staurosporine (Sta) for 1 hr, then continuously co-cultured with various doses of vglycin for 18 hr. The bar graph (top) indicates the apoptotic rate as determined by flow cytometry analysis. After that, the cells were collected and subjected to immunoblotting analysis with the indicated antibodies (down) (**H**). The data are presented as mean ± SEM values and are representative of three independent experiments. *,**,***p < 0.05, 0.01, 0.001 *vs.* the basal control. ^#,##^p < 0.05, 0.01 *vs.* the positive control.

To elucidate the mechanisms of increased replication of β-cells during chronic vglycin treatment, we used the rat pancreatic β-cell line INS-1 832/13 as an *in vitro* model. The distribution and localization of vglycin in INS-1 832/13 cells were detected by confocal analysis. As shown in Fig. 7A, vglycin (red) enters the cytoplasm within 5 min of administration, and most of the vglycin enters the cell nuclei within 60 min, suggesting its physiological potential. Flow cytometry was used to investigate the cell cycle. Cell proliferation was detected using cell count kit 8. As indicated in Fig. 7B, cell cycle analysis revealed that vglycin accelerates INS-1 cells from S phase to G2/M phase in a dose-dependent manner. Vglycin facilitated the rate of cell division, thereby indicating an increased rate of proliferation rate. After 24 hr, 48 hr and 72 hr of vglycin treatment, absorbance values were used to estimate cell numbers. Vglycin also promoted cell proliferation in a dose-dependent manner, as shown in Fig. 7C. These results are consistent with those of the cell cycle assay. We next investigated the implicated mechanisms by which vglycin stimulates INS-1 proliferation by determining if vglycin activates the IR *in vitro* as observed *in vivo*. As shown in Fig. 7D, vglycin activated the IR in 5 min, and the phosphorylated status was maintained for a long period (240 min) with the stimulation of vglycin. The downstream kinases of IR, Akt and Erk were also subsequently activated. To determine if vglycin is an essential factor in the process, vglycin was added for 5 min and then removed. Samples were collected at 0 min, 60 min and 120 min. Western blot results indicated that the levels of phosphorylated IR, Akt and Erk decreased after removal of vglycin (Fig. 7E). To further investigate the roles of IR in vglycin-mediated INS-1 growth, the IR/IGF-I receptor-specific inhibitor Ag1024 was used to pretreat INS-1 cells before adding vglycin. As shown in Fig. 7F, inhibition of IR potently blocked the vglycin-mediated activation of IR and its downstream kinases. In addition, cell proliferation was also significantly suppressed in Ag1024-treated INS-1 cells (Fig. 7G). In summary, we propose that vglycin contributes to the proliferation of pancreatic β-cells via the IR/Akt/Erk pathway.

The *in vivo* data indicate that vglycin protects islets cells from apoptosis. To confirm the anti-apoptotic effect of vglycin, INS-1 was pretreated with staurosporine (400 nM) for 1 hr and, after removal of the staurosporine, continuously co-cultured with various doses of vglycin for 18 hr. The cells were then analyzed by flow cytometry. As shown in Fig. 7H (top), pretreatment with staurosporine for 1 hr increased the percentage of apoptotic cells from 0.5% to 16% of total cells; this percentage was distinctly reduced to 10.8%, 7.65% and 3.4% when cells were also administered 50 nM, 100 nM or 300 nM vglycin, respectively. A representative result of the apoptosis analysis is shown in Supplementary Fig. 5. The immunoblot analysis was performed in triplicate. A representative western blot result is shown in Fig. 7H. The amount of cleaved PARP was reduced in the vglycin-treated groups. The levels of Pdx-1 and phospho-Akt (ser473) were significantly elevated in the vglycin-treated groups (Fig. 7H). Akt and Pdx-1 are two key factors in the survival and function of pancreatic β-cells; thus, vglycin contributes to the survival of β-cells by elevating the levels of Pdx-1 and phospho-Akt. Taken together, these results indicate that vglycin promotes INS-1 proliferation via a mechanism that directly targets IR and protects β-cells from apoptosis induced by cytotoxic substances *in vitro*.

## Discussion

Epidemiological and clinical evidence indicate that β-cell dysfunction and destruction occur frequently in metabolic diseases such as obesity and diabetes^1^. Targeting and restoring impaired β-cell function is an attractive concept for combating diabetes and its complications^11^,^14^. In the present study, we isolated a novel bioactive peptide, vglycin, from pea (*Pisum sativum* L.) seeds and explored its possible activity in pancreatic β-cells. Our pharmacological and animal models consistently revealed that vglycin has obvious physiological effects on the proliferation and survival of pancreatic β-cells, in agreement with studies demonstrating that a soybean diet is beneficial to diabetics^41^,^42^. Our data support a clear effect of vglycin on regulating β-cell preservation by initiating a self-replication program.

To characterize the effect of vglycin on β-cells *in vivo*, T1DM SD rats with a clear metabolic background were treated with vglycin. BW, food intake and serum plasma glucose levels were significantly decreased in the vglycin-treated group, while the wet pancreas mass, β-cell area, β-cell mass and number of islets were increased. These results are consistent with our finding that cleaved caspase-3 levels, PARP content and the percentage of TUNEL-positive cells in the islets were lower in the vglycin-treated group than the control group. Conversely, the Ki67/PCNA-positive area and the expression of Erk, Nkx6.1 and MafA were slightly increased in the vglycin-treated group. Immunofluorescence directly indicated the effect of vglycin on the proliferation and preservation of β-cells. Given the ability of vglycin to promote β-cell survival under STZ stress conditions, we are optimistic that vglycin could be used therapeutically to promote β-cell survival in the context of multiple stress conditions.

Adulthood diabetic mouse models were generated by feeding mice with a HFD over a long period with or without two low intraperitoneal doses of STZ. Using these models, we demonstrated *in vivo* that vglycin contributes to the proliferation and survival of β-cells challenged by HFD or HFD and STZ. Mice treated with vglycin exhibited decreases in BW and Lee’s index, possibly due to decreased energy intake, and low levels of FPG and random-fed glucose should be associated with improved insulin sensitivity and glucose utilization. Because this metabolic improvement is indirectly beneficial to the survival of β-cells, we speculated that β-cells were improved by vglycin treatment. Indeed, we demonstrated that after chronic vglycin treatment, secretion of insulin and glucagon were upregulated and downregulated, respectively, in both vglycin-treated groups after glucose loading. Exposure to an appropriate dose of IL-1β/6 for a limited period is beneficial for β-cells. Accordingly, we observed a decline in plasma IL-1β levels in the HFD + STZ + Vg group and increased pancreatic IL-6/1β expression in both vglycin-treated groups. Histological analysis revealed that the pancreas mass was greater in both vglycin-treated groups than the control groups. The β-cell areas, β-cell mass and number of islets in the HFD + STZ + Vg group were slightly increased. Double immunofluorescence staining revealed the ratios of Ins^+^ cells to Glu^+^ cells, Ki67^+^ and Ins^+^ double-positive cells to total ß-cells, Pdx-1^+^ and Ins^+^ double-positive cells to total ß-cells, and TUNEL^+^ cells to total ß-cells in each group. These ratios indicated that chronic vglycin treatment contributes to the proliferation and conservation of β-cells. In both vglycin-treated groups, activated IR, Akt and Erk, which are critical regulators of β-cell survival and proliferation, were prominently upregulated, consistent with the elevated expression of the cell proliferation marker Ki67. The β-cell markers Pdx-1 and MafA and the mature β-cell markers Nkx6.1and FoxO-1 were strikingly upregulated in both vglycin-treated groups. However, the dedifferentiation marker NGN3 was negatively regulated in both vglycin-treated groups. Although previous studies have indicated that FoxO-1 is a negative regulator of β-cell proliferation^38^,^43^, the present study indicates that vglycin not only promotes the proliferation of β-cells but also contributes to their maintenance. Consistent with the immunofluorescence data shown in Fig. 5A,E, cleaved caspase-3 levels were significantly lower in both vglycin-treated groups than in the corresponding controls. To substantiate these findings, the rat pancreatic β-cell line INS-1 832/13 was directly exposed to vglycin. Consistent with the *in vivo* data, our *in vitro* data indicated that vglycin promotes β-cell proliferation and contributes to the preservation of INS-1 when challenged by staurosporine.

We previously demonstrated that vglycin ameliorates diabetes in a T2DM Wistar rat model by improving insulin sensitivity and glucose tolerance^21^. We initiated the present study by probing the potential metabolic action of vglycin in preserving pancreatic β-cells under metabolic stress. We discovered that vglycin responds to the metabolic signals produced by STZ or overnutrition and generally contributes to the proliferation and survival of β-cells in both juvenile T1DM SD rats and middle-aged T2DM C57BL/6 mice. Importantly, this effect was accompanied by a significant attenuation of peripheral inflammation with downregulation of proinflammatory mediators such as IL-1β and a beneficial switch to IL-1β/6 expression. Consistent with the *in vivo* observations, our *in vitro* data also indicated that IR plays a key role in mediating the vglycin-induced proliferation of β-cells, as the effect is abrogated in INS-1 832/13 cells pretreated with an inhibitor of IR. Recent studies have indicated that β-cells lose their characteristics and transdifferentiate into other cell types under metabolic stresses such as aging, overnutrition and STZ. Our results also indicate that vglycin can maintain the characteristics of β-cells by upregulating β-cell markers such as Pdx-1 and MafA as well as FoxO-1 and Nkx6.1 while downregulating the ancestry marker NGN3.

**Figure 8:**
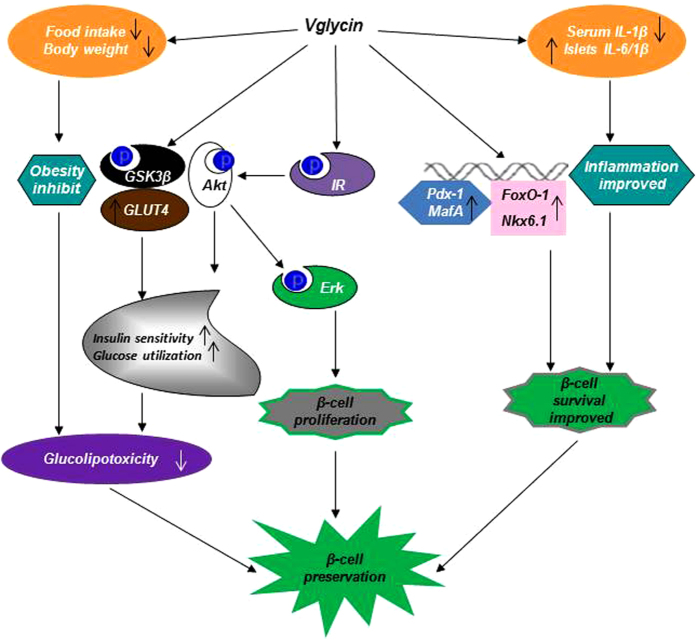
Schematic of the proposed role of vglycin in the preservation of diabetic β-cells. We propose that vglycin directly promotes the proliferation of β-cells via the IR/Akt/Erk pathway. We also further postulate that vglycin indirectly promotes the preservation of β-cells by attenuating the stress of glucolipotoxicity and inflammation, which may consequently protect β cells from apoptosis.

As outlined in Fig. 8, we propose that the acute and chronic *in vivo* effects of vglycin on β-cells are not solely due to decreased blood glucose or BW. The enhanced insulin sensitivity and glucose utilization observed in vglycin-treated animals may help decrease glucolipotoxicity and relieve secretion pressure in β-cells, although other mechanisms may be involved, such as the ability of vglycin to attenuate peripheral inflammation and the capacity of cytokines to impair β-cells. Collectively, the present study reveals a previously unrecognized role of vglycin in the preservation of β-cells and points to a potential role of this soybean peptide in the treatment of diabetes, although the precise molecular mechanisms remain to be elucidated. The positive effects of vglycin on β-cells should inspire substantial interest in vglycin as a potential treatment for diseases such as diabetes and obesity.

## Methods and Materials

### Chemicals and reagents

Both vglycin and aglycin were purified and characterized as previously described by our laboratory. The preparation protocol and the primary amino acid structure are shown in supplementary Fig. 1A and 1B. Analysis by high-performance liquid chromatography (HPLC) demonstrated that the purity of vglycin (or aglycin) was greater than 95%. A portion of the vglycin was labeled using a rhodamine labeling kit according to the instructions provided by the manufacturer (Pierce Biotechnology). The antibodies used for immunoblotting, immunohistochemistry and immunofluorescence are listed in Supplementary Table 1. All other chemicals were purchased from Sigma Chemical Co. or Beijing Chemical Reagents Company unless otherwise noted.

### Animal experiments

Male Sprague Dawley (SD) rats and male C57BL/6 mice at approximately 6 weeks of age were obtained from the Animal Experimental Center of Wuhan University (ABSL-3). All animals were housed in a standard, pathogen-free animal facility in the Animal Experimental Center of Wuhan University (ABSL-3) with a 12-hr light/dark cycle at 22-24°C, and animals were given free access to food and water for the duration of the experiment except during fasting tests (no more than 16 hr) as required. Rats were maintained on normal chow (10% kcal fat, Medicience Ltd, China) after weaning. A standard HFD (45% kcal fat, Medicience Ltd, China) was used when mice were two months old. Body weight and food (or energy) intake were measured using a dedicated scale. Mouse GTT was performed via intraperitoneal (i.p.) injection and oral administration (o.a.) of glucose at 1.5 g/kg body weight. ITT was performed in 3-hour fasting mice via i.p. injection of human recombinant insulin (Nova Nordisk) at a dose of 1U/kg body weight. Glucose levels in blood collected from the tail vein were determined using a One Touch^®^ Ultra^®^ Blood Glucose Test System Kit (Lifespan Company, USA).

Rats with similar FPG and body weight were randomly assigned to the control group, STZ group or STZ + Vglycin group. To induce experimental Type 1 diabetes with destruction of the β-cell population, rats in the STZ and STZ + Vglycin groups received 4 intraperitoneal injections of STZ at a dose of 45 mg/kg body weight after a 15-hr fasting period, as indicated in supplementary Fig. 2A. Rats in the STZ + Vglycin group were orally administered vglycin dissolved in saline at a dose of 80 μg/g body-weight/day, while the other two groups were treated with the same volume of saline.

Mice were randomly assigned to 5 groups designated Chow, HFD, HFD + Vglycin, HFD + STZ and HFD + STZ + Vglycin. Mice in the Chow group were fed normal chow for the duration of the experiment, while the other four groups were fed standard HFD from the age of 2 months. To induce experimental Type 2 diabetes with impaired insulin sensitivity and partial β-cell dysfunction, mice in the HFD + STZ and HFD + STZ + Vglycin groups received multiple low doses (25 mg/kg body weight) of STZ intraperitoneal injections at the age of 20–21 weeks, as indicated in supplementary Fig. 2B. Mice in the HFD + STZ + Vglycin and HFD + Vglycin groups were orally administered vglycin dissolved in saline at a dose of 20 μg/g body-weight/day, while the other groups were treated with the same volume of saline.

Animals were cared for in accordance with the National Institutes of Health Guidelines for Animal Care. All experimental procedures were reviewed and approved by the Institutional Animal Care and Use Committee at the Animal Experimental Center of Wuhan University (ABSL-3) (Approval number: 2013112). All experiments were carried out in accordance with the approved guidelines.

### Immunoblotting and histological analysis

Immunoblotting was performed as previously described. Briefly, proteins were extracted from the indicated tissues (liver, muscle and pancreas) and INS-1 cells in the presence of a cocktail of protease inhibitors and phenylmethylsulfonyl fluoride (PMSF) (Roche Applied Science), resolved and separated by 12–15% SDS-PAGE, and transferred to PVDF membranes. The membranes were then probed with the primary antibodies listed in supplementary Table 1, followed by incubation with the corresponding horseradish peroxidase-conjugated secondary antibodies. The protein bands were visualized with enhanced chemiluminescence reagents (Thermo Fisher Scientific, USA), detected using the ECL system, and quantified using NIH ImageJ software.

Tissues were post-fixed in 4% PFA for 24 hr and sectioned after embedding in paraffin. The sections were prepared and stained with H&E using standard procedures. All slides in the current study were examined under a Nikon ECLIPSE Ci biological microscope, and images were captured with a Nikon DS-U3 color digital camera unless otherwise noted.

### Immunohistochemistry and immunofluorescence

Conventional immunohistochemistry was conducted on fixed wax-embedded pancreatic sections as previously described. Briefly, isolated pancreata were fixed in 4% PFA at 4°C overnight. Tissues were then embedded in paraffin, and 2-μm sections were prepared and mounted on glass slides. The sections were then immunostained with rabbit anti-proliferating cell nuclear antigen (Ki67 and PCNA) antibodies. The immunofluorescence TUNEL staining (In Situ Cell Death Detection Kit, Roche Diagnostics Corp.) was performed to detect apoptotic cells. For insulin positivity, the islet insulin immunoreactive cross-sectional area and total pancreatic area were analyzed, calculated using NIH Image J software, and expressed as the percentage of β-cell area relative to the total pancreatic area.

Immunofluorescence staining was performed according to standard protocols using the antibodies and dilutions listed in supplementary Table 1, followed by incubation with the corresponding secondary antibodies conjugated with Alexa 488 (green) or Alexa 594 (red) fluorescent dyes purchased from Invitrogen. 4′,6-Diamidino-2-phenylindole (DAPI) was used to label cell nuclei. For proliferation analysis, Ki67-positive β-cells were counted in pancreatic sections costained for insulin and expressed as a percentage of Ki67 and insulin double-positive cells per total ß-cell nuclei. Apoptosis was evaluated on pancreatic paraffin sections using immunofluorescence TUNEL staining. For apoptosis quantification, the number of intra-islet TUNEL positive cells per total ß-cell nuclei was determined. Quantification of Pdx-1 was performed in pancreatic sections double stained for insulin and Pdx-1 by counting the number of Pdx-1 and insulin double-positive cells per total ß-cell nuclei in a merged red and green image. To estimate the pancreatic α-cell population, the total number of intra-islet glucagon-positive cells was counted from several pancreatic sections immunostained with anti-glucagon antibody.

### Enzyme-linked immunosorbent assay

Mouse TNF-α, IL-6 and IL-1β ELISA kits (Neobioscience, Shenzhen, China) and mouse insulin, C-peptide and glucagon ELISA kits (RayBiotech, USA) were used. The levels of these inflammation factors and hormones in both sera and tissues were quantified by enzyme-linked immunosorbent test according to the manufacturer’s instructions.

### Confocal microscopy

Confocal analysis was performed as previously described. Briefly, vglycin was labeled using a rhodamine labeling kit (Pierce Biotechnology, USA), added to 24-well plates and co-cultured with INS-1 at a final concentration of 100 nM. At the indicated time points, the medium was removed, and INS-1 was fixed in 4% paraformaldehyde (PFA) in PBS for 10 min at room temperature and then permeabilized in 0.5% Triton X-100 PBS for another 10 min. The cells were then preblocked with 5% FBS for 1 h, followed by incubation with DAPI for 5 min. Finally, optical images were acquired using an inverted system microscope (Olympus IX71).

### Cell preparation and treatment

INS-1 832/13 cells were obtained from American Type Tissue Culture Collection (passage 40–60) and cultured under an atmosphere of 5% CO_2_ and 95% O_2_ at 37 °C in RPMI 1640 medium (Gibco Chemical, USA) containing 10% (v/v) fetal bovine serum (FBS), 11.1 mM glucose, 100 U/mL penicillin, and 0.1 mg/mL streptomycin. INS-1 832/13 cells were treated or pretreated with vglycin after 12–15 hr in culture in RPMI 1640 serum starvation medium containing 2% (v/v) FBS. For inhibition tests, INS-1 was pretreated with 0.2 μM AG1024 (Merck Biosciences) for 4 hr and exposed to serum starvation medium containing 300 nM vglycin for 24 hr. For the anti-apoptosis tests, INS-1 was first starved by incubation in starvation medium overnight and then pretreated with 400 nM staurosporine (Beyotime Institute of Biotechnology, China) for 1–4 hr. The cells were then continuously co-cultured with various doses of vglycin for 18 hr.

### Cell proliferation test and flow cytometry

Cell proliferation was determined using either the Cell counting kit 8 (CCK8) (Dojindo, Japan) or flow cytometry (Beckman, USA). For the CCK8 assay, the cell number was determined using a microplate reader based on absorbance values. The cell cycle stages were analyzed based on propidium iodide (Beyotime Institute of Biotechnology, China) staining of DNA using flow cytometry. Relative cell apoptosis was measured by flow cytometry using Annexin V-FITC Apoptosis Detection Kit (Dojindo, Japan). Flowjo software was used for analysis.

### Statistical analysis

Comparisons between two groups were tested using an unpaired Student’s t-test (GraphPad Software), and differences among several groups were tested using a post-hoc Duncan’s test (SPSS Version 16.0, USA). All data were normally distributed. Statistical tests and sample sizes of each data set were deemed appropriate. Randomization was used in animal experiments. However, the researchers were often not blinded to the distribution of treatment groups when performing experiments and data assessment. Data are presented as the mean ± s.e.m. and were plotted using GraphPad Software. Results were considered significant at p < 0.05.

## Additional Information

**How to cite this article**: Jiang, H. *et al.* The Soybean Peptide Vglycin Preserves the Diabetic β-cells through Improvement of Proliferation and Inhibition of Apoptosis. *Sci. Rep.* **5**, 15599; doi: 10.1038/srep15599 (2015).

## Supplementary Material

Supplementary Information
