# Supplementary material for: The Soybean Peptide Vglycin Preserves the Diabetic β-cells through Improvement of Proliferation and Inhibition of Apoptosis
Source: Sci Rep. 2015 Oct 29;5:15599. doi: 10.1038/srep15599 (PMC4625148; doi:10.1038/srep15599)

Supplementary Information

The Soybean Peptide Vglycin Preserves the Diabetic β-cells through Improvement of Proliferation and Inhibition of Apoptosis

Hua Jiang[1 2 6 #], Yuxing Tong[1 6], Dongjing Yan[3], Shaohui Jia[4], Claes-Goran Ostenson[5] and Zhengwang Chen[1 #]

[1]Key Laboratory of Molecular Biophysics of Ministry of Education, School of Life Science and Technology, Huazhong University of Science and Technology, Wuhan 430074, P.R. China.

[2]Institute of Immunology and the CAS Key Laboratory of Innate Immunity and Chronic Disease, School of Life Sciences and Medical Center, University of Science and Technology of China, Hefei 230027, China.

[3]Department of Biochemistry and Molecular Biology, Hainan Medical College, Haikou 571199, China;

[4]College of Health Science, Wuhan Sports University, Wuhan 430079, P.R. China.

[5]Department of Molecular Medicine, Karolinska Hospital, Stockholm, Sweden.

[6]These authors contributed equally to this work.

[#]Correspondence: zwchen@hust.edu.cn (Z.C.); jianghub@ustc.edu.cn (H.J.).

Supplementary table 1.
Antibodies used for Western blotting and immunofluorescent staining.

Primary Antibody	Clone	Company	Catalog No.	Dilution
Nkx 6.1	N-15	Santa Cruz	sc-15027	1:1000(WB)
MafA	F-6	Santa Cruz	sc-390491	1:1000(WB)
FoxO1	Polyclonal	ABclonal	A2934	1:1000(WB)
Phospho-IR(Tyr1146)	Polyclonal	Cell Signaling	#3021	1:1000(WB)
Phospho-IR(Tyr1150/1151)	19H7	Cell Signaling	#3024	1:1000(WB)
IR	4B8	Cell Signaling	#3025	1:1000(WB)
Phospho-GSK3α/β	Ser21/9	Cell Signaling	#9331	1:1000(WB)
GLUT4	1F8	Cell Signaling	#2213	1:1000(WB)
Akt	Polyclonal	Cell Signaling	#9272	1:1000(WB)
Phospho-Akt(Ser473)	D9E	Cell Signaling	#4060	1:1000(WB)
Phospho-Akt(Thr308)	244F9	Cell Signaling	#4056	1:1000(WB)
PARP	Polyclonal	Cell Signaling	#9542	1:1000(WB)
β-actin	H-196	Santa Cruz	sc-7210	1:1000(WB)
Erk1/2	Polyclonal	Cell Signaling	#9102	1:1000(WB)
Phospho-Erk1/2	Polyclonal	Cell Signaling	#9101	1:1000(WB)
NGN3	Polyclonal	ABclonal	A2772	1:1000(WB)
H2A	Polyclonal	Santa Cruz	Sc-86470	1:1000(WB)
Caspase-3	Polyclonal	Cell Signaling	#9662	1:1000(WB)
GAPDH	FL-335	Santa Cruz	sc-25778	1:1000(WB)
Glucagon	Polyclonal	Cell Signaling	#2760	1:200(IF)
PCNA	Polyclonal	Santa Cruz	sc-7907	1:40(IHC)
Insulin	L6B10	Cell Signaling	#8138	1:200(IF)<br>1:100(IHC)
Pdx1	H-140	Santa Cruz	sc-25403	1:1000(WB)<br>1:50 (IF)
Ki67	D3B5	Cell Signaling	#9129	1:1000(WB)<br>1:100(IF)<br>1:100(IHC)
Secondary antibody	Conjugate	Company	Catalog No.	Dilution
Anti-mouse IgG	HRP	Cell Signaling	#7076	1:20000(WB)
Anti-rabbit IgG	HRP	Cell Signaling	#7074	1:20000(WB)
donkey anti-goat IgG	HRP	Santa Cruz	sc-2020	1:10000(WB)

Supplementary table 2.
Diet formula and fat composition.

Element / Diet	Protein (kcal%)	Carbohydrate (kcal%)	Fat (kcal%)	Company (China)	Catalog No.
Normal Chow	20	70	10	Medicience Ltd.	MD12031
High Fat Diet	20	35	45	Medicience Ltd.	MD12032

Fatty acid / Diet	Polyunsaturated (%)	Monounsaturated (%)	Saturated (%)	Company (China)	Catalog No.
Normal Chow	38.6	32.7	28.7	Medicience Ltd.	MD12031
High Fat Diet	19.3	40.4	40.3	Medicience Ltd.	MD12032

Fat type / Diet	Soybean Oil (gm)	Lard (gm)	Company (China)	Catalog No.
Normal Chow	25	20	Medicience Ltd.	MD12031
High Fat Diet	25	177.5	Medicience Ltd.	MD12032

Supplemental Figure 1. Related to the methods and materials.

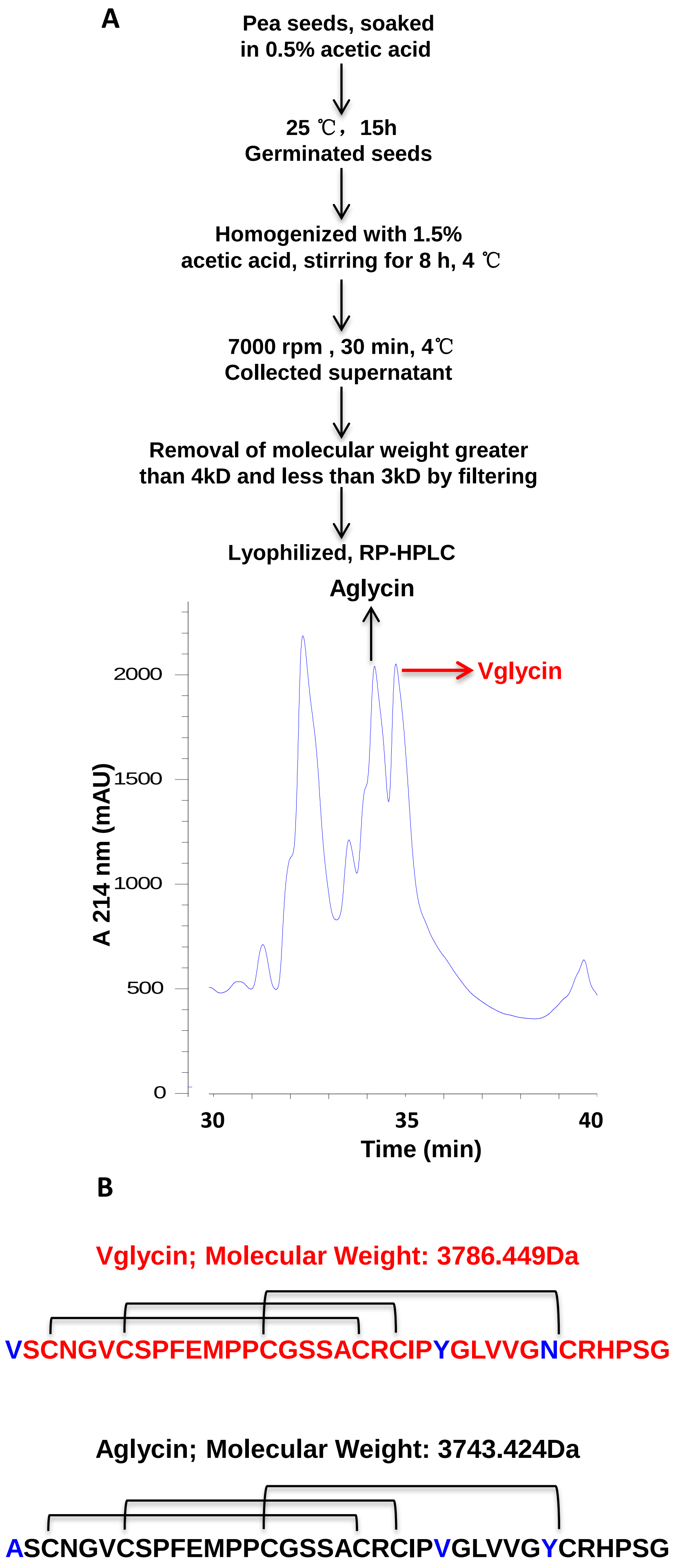

Supplemental Figure 2. Related to the methods and materials.

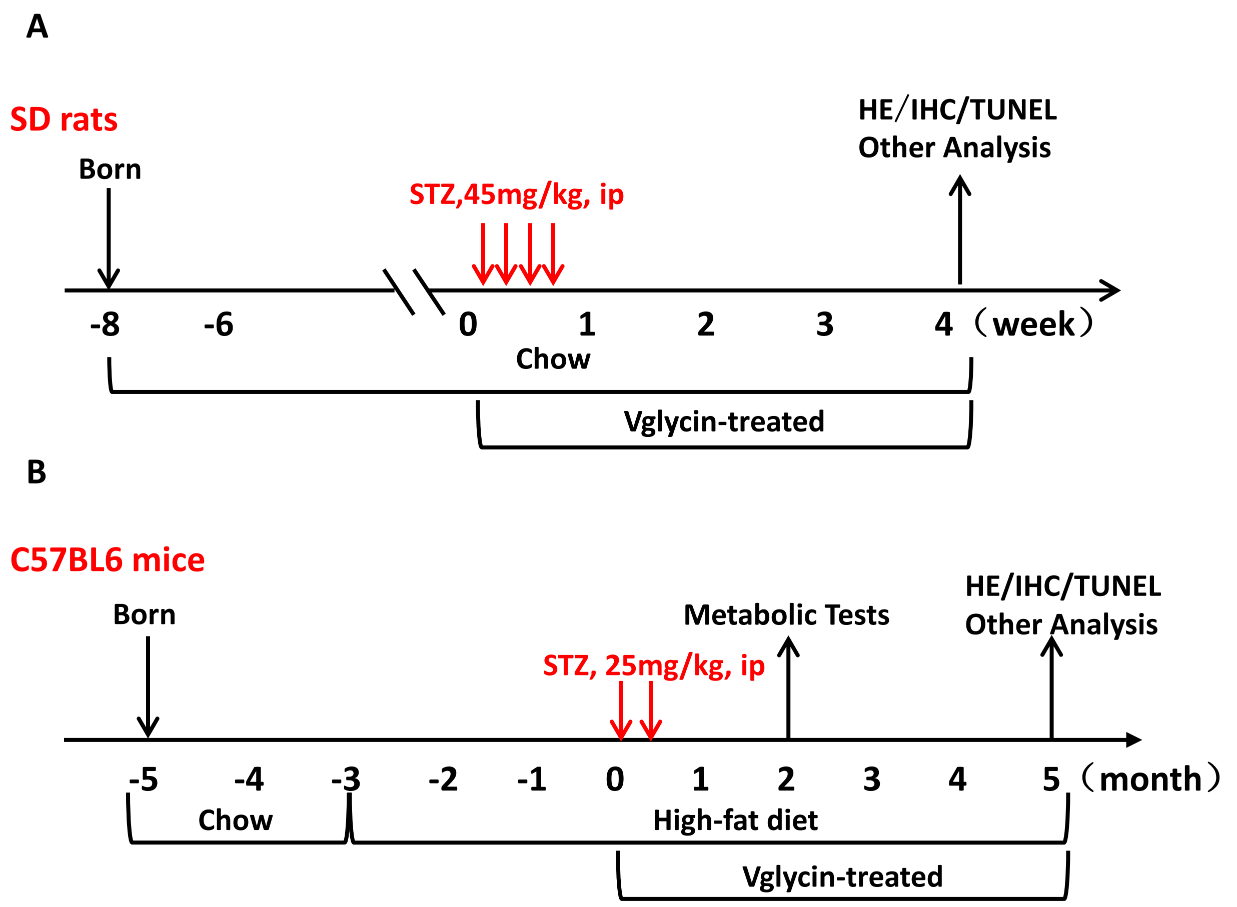

Supplemental Figure 3. Related with Figure 1I and 1J.

A

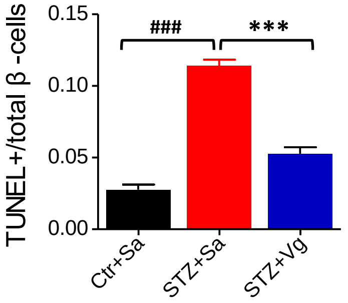

B

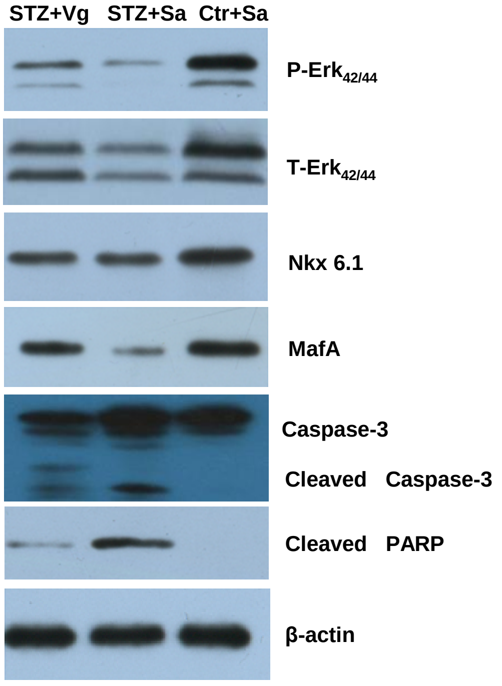

Supplemental Figure 4. Related with Figure 3G, 3H, 3J and 3K.

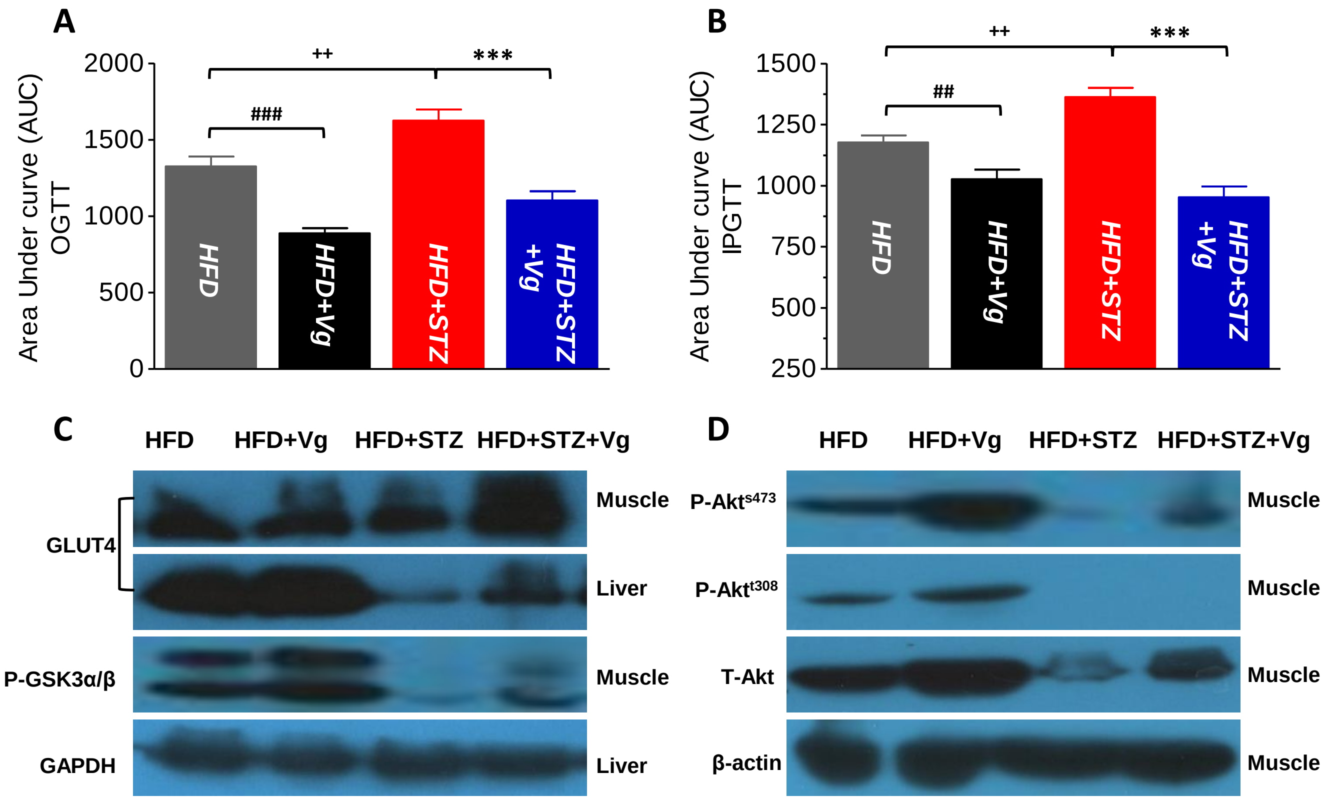

Supplemental Figure 5. Related with Figure 6A, 6B and 6C.

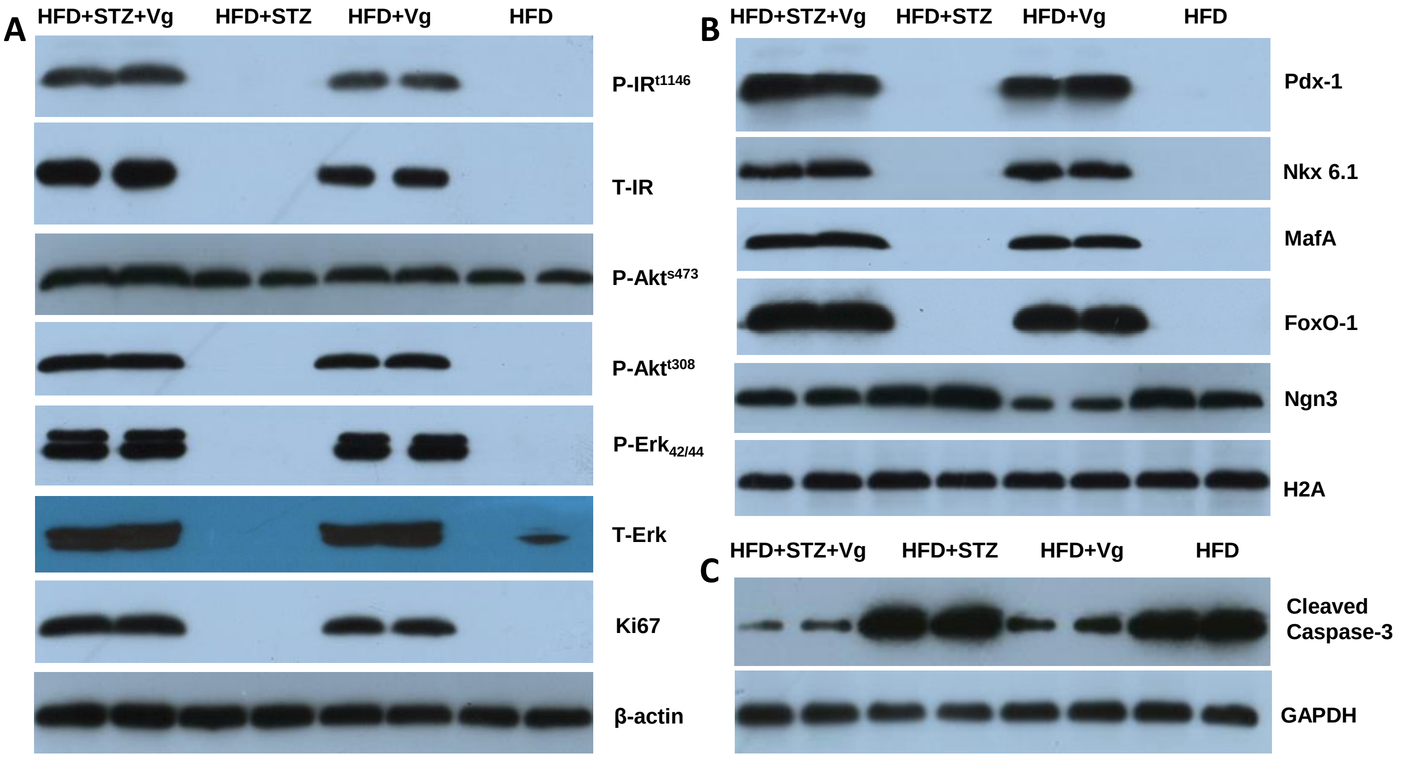

Supplemental Figure 6. Related with Figure 1J, 7D, 7E, 7F and 7H.

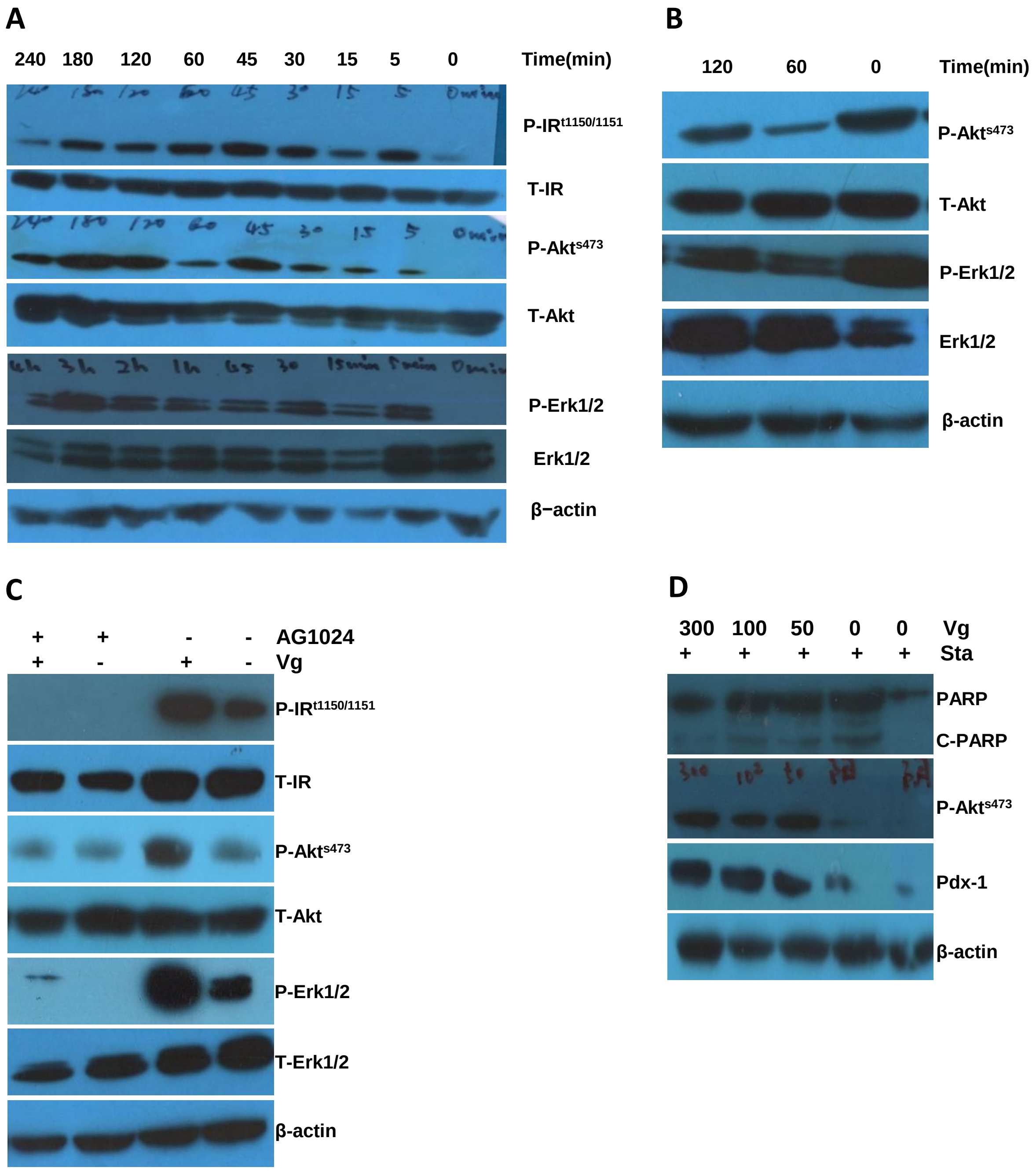

Supplement: Supplementary Information [file srep15599-s1.pdf]
